# Postpolypectomy fever in patients with serious infection: a report of two cases

**DOI:** 10.1186/s12876-022-02218-9

**Published:** 2022-03-29

**Authors:** Wang Jing, Li Qinghua, Yang Zhiwen

**Affiliations:** 1 Department of Gastroenterology, Songjiang District Central Hospital, Shanghai, China; 2 Department of Pharmacy, Songjiang District Central Hospital, Shanghai, 201600 China

**Keywords:** Postpolypectomy fever, Serious infection, Diagnose, Therapy, Patient

## Abstract

**Background:**

Postpolypectomy fever (PPF) is a rare complication in patients after colonoscopy. Because of the absence of evidence of microperforation and abdominal tenderness, patients with PPF usually present mild clinical symptoms with a good prognosis.

**Case presentation:**

In this study, all patients who underwent colonoscopic examination in our hospital between January 2019 and December 2019 were enrolled. Of these, two patients developed PPF after polypectomy, exhibiting serious infection without definitive fever foci. One patient experienced rapidly aggravated type 1 respiratory failure and abnormal hepatic function, which were attributed to colonoscopy-associated infection. After active antibiotic therapy, both patients were discharged without any complications.

**Conclusions:**

In summary, our study provides novel insights into patients with PPF who develop serious infections with life-threatening complications.

## Background

Colonoscopy is widely used in clinical practice, although serious complications may result from colonoscopic polypectomy [1, 2]. These serious complications are inherent to the procedure and occur at low incidence during colonoscopy. Hemorrhage and perforation, the most feared complications, occur in ≤ 0.3% and 0.3–0.6% [3, 4], respectively. Postpolypectomy electrocoagulation syndrome (PPCS), a rare complication, ranges from 0.07 to 1.0% [3, 4].

Here, we report the cases of two patients who developed PPF after colonoscopy, and experienced new-onset fever without localized peritoneal signs or definitive fever foci. Aggravated serious infectious symptoms were present with a high fever up to 39.0 °C in 1–2 h after operation, and the patients received further therapy in the general intensive care unit (GICU). As reported previously, patients with PPF generally present mild clinical symptoms with good prognosis. To our knowledge, this is the first report of patients with PPF developing serious infections with life-threatening complications. Additionally, PPF is easily overlooked in clinical practice, owing to the absence of typical peritoneal irritation and definitive fever foci. Thus, our report should aid in timely diagnosis and appropriate therapy for patients with PPF.

## Case presentation

### Case selection, procedures, and definitions

In 2019, 12,000 patients underwent colonoscopic examination in our hospital, and approximately 2000 patients with gastrointestinal polyps received painless endoscopic treatment. These cases of colonoscopies were performed in the outpatient and inpatient setting. All colonoscopies were elective, not urgent. Patients who received polypectomy were admitted to the hospital for about 2–3 days. Two patients after colonoscopy met the inclusion criteria with a high fever up to 39.0 °C in 1–2 h, and the leukocytes, C-reactive protein (CRP) and procalcitonin (PCT) increased significantly with signs of infection [5–7]. They were a health state at admission, without any signs of fever, abdominal pain, cough, frequent urination, infection or other discomfort. The results of routine analyses of the blood, urine and feces were normal, and no signs of infection were observed on chest、abdominal CT. The patients developed serious infections after polypectomy during hospitalization. Physical and radiographic examination did not show evidence of perforation, hemorrhage, abdominal tenderness or localized peritoneal inflammation. No evidence of other explainable fever foci other than colonoscopic polypectomy was identified.

Patients with postpolypectomy bleeding, microperforation, abdominal tenderness, localized peritoneal inflammation and infection associated with definitive fever foci other than colonoscopic polypectomy were excluded.

All colonoscopic polypectomies were performed with standard colonoscopes (CF-H260AL; Olympus Optical Co., Ltd., Tokyo, Japan). Patients were slowly intravenously injected with propofol. Patients who received polypectomy operation were admitted to the hospital for about 2–3 days.

### Patient 1

**Fig. 1:**
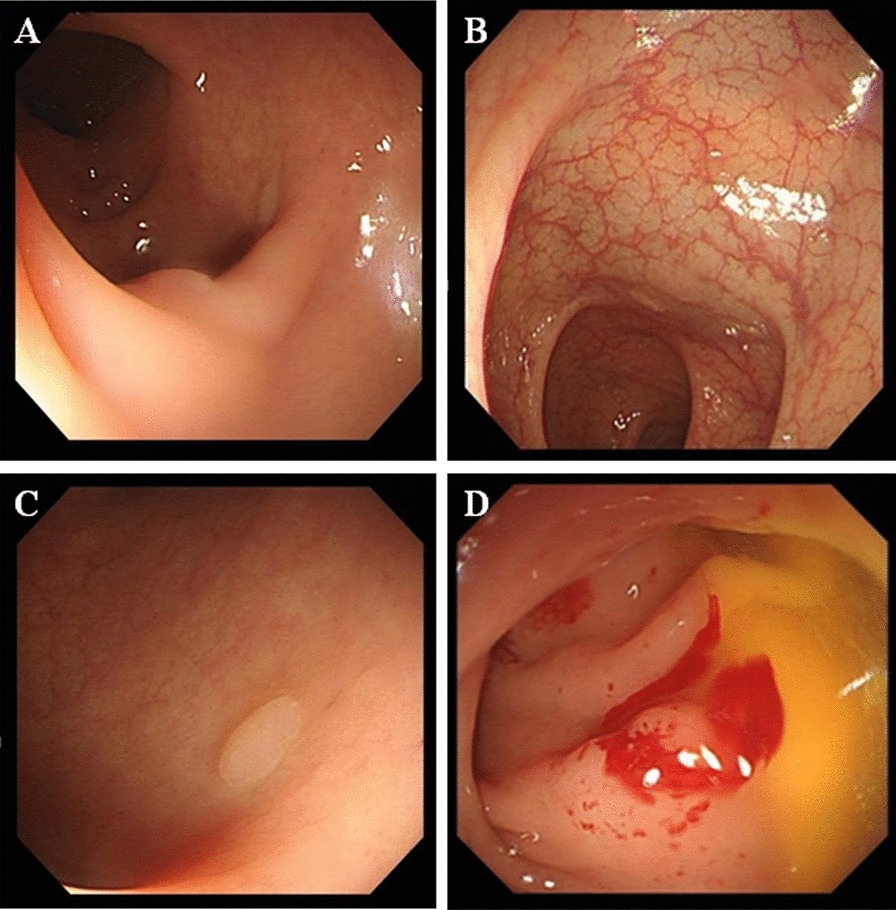
Colonoscopic examination of a 50-year-old man. **A** A flat polyp with a diameter of 3 mm in the sigmoid colon. **B** Two flat polyps with diameters of 3–5 mm in the sigmoid colon. **C** A flat polyp with a diameter of 4 mm in the rectum; **D** Rectal biopsy

**Fig. 2:**
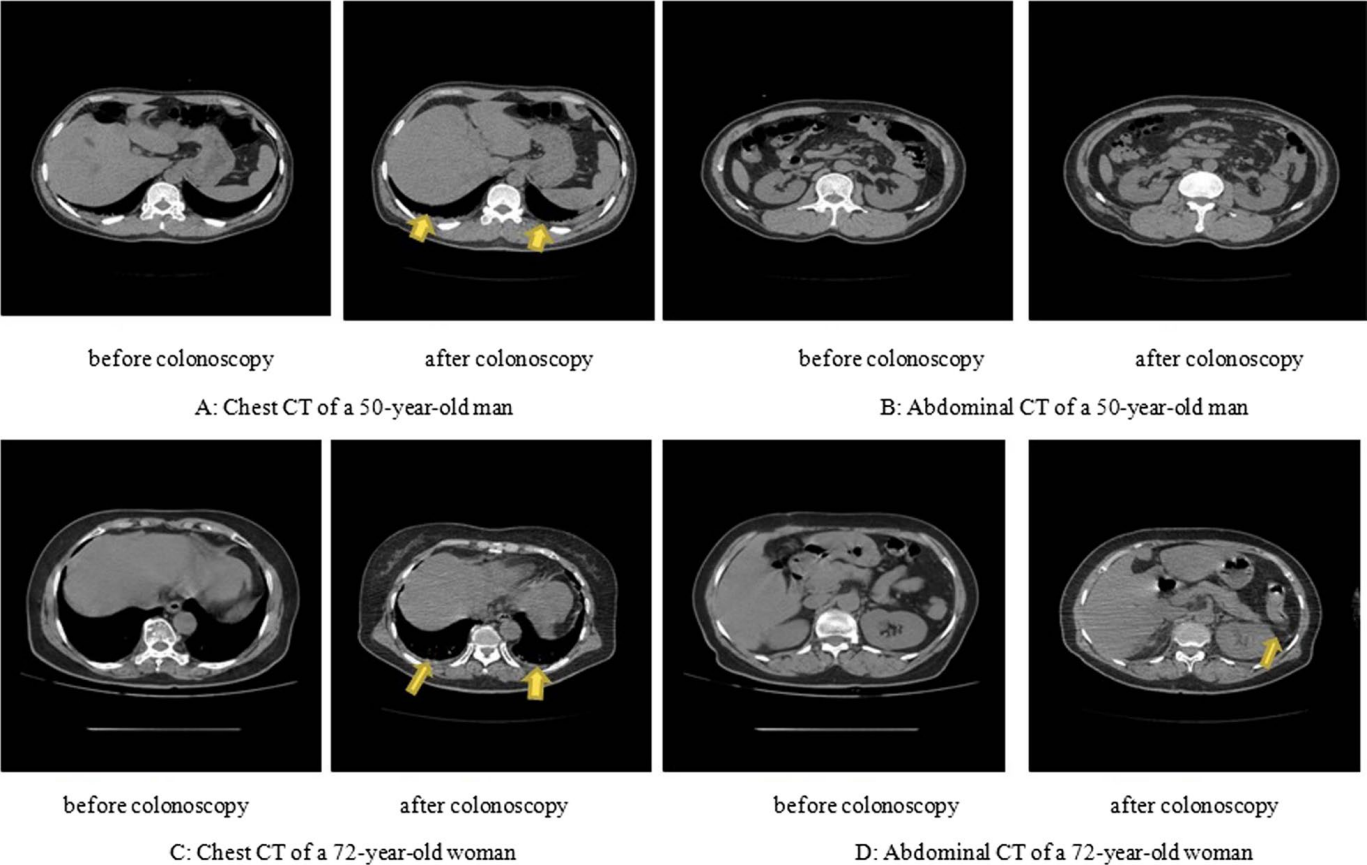
chest and abdominal CT before and after colonoscopy. **A** Chest CT of a 50-year-old man. Normal CT before colonoscopy, but abnormal CT with pleural effusion after colonoscopy. **B** Abdominal CT of a 50-year-old man. Normal CT before and after colonoscopy. **C** Chest CT of a 72-year-old woman. Normal CT before colonoscopy, but abnormal CT with pleural effusion after colonoscopy. **D** Abdominal CT of a 72-year-old woman. Normal CT before colonoscopy, but abnormal CT with exudation in pancreas tail after colonoscopy

The first case was in a 50-year-old man without a notable past history, who was diagnosed with multiple colorectal polyps. Four polyps were found: three flat polyps with a diameter of 2–5 mm in the sigmoid colon (Fig. 1A, B) and a flat polyp with a diameter of 4 mm in the rectum (Fig. 1C). He underwent colonoscopy with cold biopsy of a 4 mm rectal polyp (Fig. 1D), and the colorectal polyps were confirmed pathologically. Two hours after the operation, the patient developed a high fever up to 39.4 °C. Laboratory examinations revealed elevated infection indices, such as PCT 44.52 ng/mL, CRP 40.48 mg/L, white blood cell count (WBC) 17.3 × 10^9^/L and neutrophils 95.4%. The result of CT scan showed pleural effusion in lung (Fig. 2 A and B). No evidence of other explainable fever foci was found, and no microorganisms were found in blood cultures. Because of the serious colonoscopy-associated infection, the patient was transferred to the GICU and treated with antibiotic combined therapy consisting of meropenem (1.0 g administered intravenously every 12 h) and metronidazole (0.5 g administered intravenously every 12 h). His fever subsided within 1 day, thus indicating that the antibiotic therapy was effective. Eventually, the patient was discharged without any complications.

### Patient 2

**Fig. 3:**
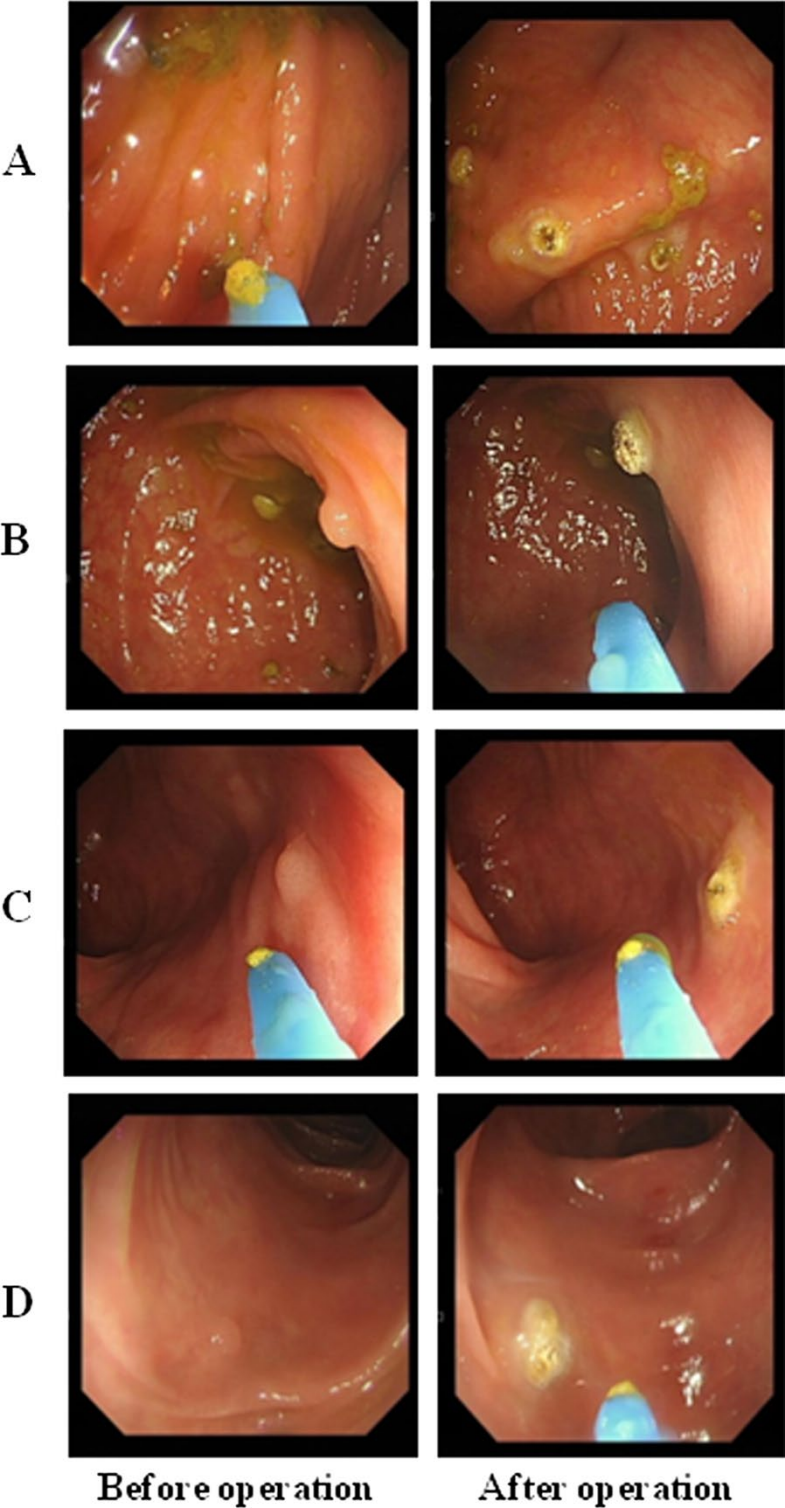
Colonoscopic examination of a 72-year-old woman. **A** Four flat polyps with diameters of 2–3 mm in the ileocecal part. **B** A 4 mm papillary polyp in the liver flexure of the colon. **C** A 3 mm flat polyp in the transverse colon. **D** Seven flat polyps with diameters of 2–5 mm in the sigmoid colon

The second case was in a 72-year-old woman with a history of hypertension and fatty liver, who underwent a colonoscopy that revealed 13 polyps: four flat polyps with a diameter of 2–3 mm in the ileocecal part (Fig. 3A), a 4 mm papillary polyp in the liver flexure of the colon (Fig. 3B), a 3 mm flat polyp in the transverse colon (Fig. 3C) and seven flat polyps with diameters of 2–5 mm in the sigmoid colon (Fig. 3D). The colorectal polyps confirmed pathologically were resected by argon plasma coagulation (APC) under the condition of strong electrocoagulation 2, 40 W APC power, and 0.8–1.2 L/min argon gas flow (APC ®2 and electric generator vio®200D; ERBE Company, Tuebingen, German). One hour after the operation, the patient suddenly developed a high fever of 39.0 °C, without cough, expectoration or abdominal pain. On the morning of the second day, the patient’s body temperature continued to rise to 40.0℃, Physical examination findings for the abdomen were negative, without evidence of intestinal perforation and hemorrhage. There was no abnormalities in routine urine examination, and no bacterial growth in blood culture examination. Laboratory examinations revealed 18.48 × 10^9^/L WBC, 96.1% neutrophils, more than 100.0 ng/mL procalcitonin, 68.24 mg/L CRP. The result of CT scan showed pleural effusion in lung and exudation in pancreas tail (Fig. 2 C and D). Of note, the patient’s illness rapidly worsened, and she was transferred to the GICU, with poor gas analysis (PH7.384, PCO_2_ 34.8 mmHg, PO_2_ 42.1 mmHg) and abnormal hepatic function results (ALT 145.9U/L, AST 256.5U/L, r-GT 215U/L). After consultation with doctors, the patient’s symptoms, including type 1 respiratory failure and abnormal hepatic functions, were attributed to a colonoscopy-associated infection with subsequent gut bacterial translocation. The patient received intravenous therapy consisting of 1.0 g of vancomycin every 12 h for 3 days, and 1.0 g of imipenem/cilastatin every 8 h for 5 days. After 2 days of therapy, her body temperature normalized. The patient's gas analysis (PH7.439, PCO_2_ 33.9 mmHg, PO_2_ 93.3 mmHg) and liver function (ALT 27.74U/L, AST 20.99U/L, r-GT 67.79U/L) also recovered normally. Eventually, she was discharged without any complications.

## Discussion and conclusions

In previously published reports, PPF has been considered a rare complication of colonoscopic polypectomy with slight clinical symptoms and good prognosis [5, 8]. To our knowledge, this study is the first report of patients with PPF presenting serious infectious symptoms leading to life-threatening complication, and rapid deterioration to type 1 respiratory failure and abnormal hepatic function. After antibiotic therapy, the patient condition rapidly recovered.

Seven patients were previously reported to develop PPF after colonoscopic polypectomy, of which four cases had a polyp diameter ≥ 2 cm, one case had a polyp 10–30 mm in diameter, and two cases had no polyps [5]. The median initial time of fever after polypectomy was approximately 7 h, and the median fever duration was approximately 9 h [5]. The seven patients with PPF had slight clinical symptoms with a good prognosis after antibiotic therapy [5]. CRP, a critical infection index, did not increase within 24 h [5]. In contrast to these cases, three exceptional findings in our study were observed. First, severe infection in patients with PPF was found, thus resulting in type 1 respiratory failure and abnormal hepatic function. Second, the patients with PPF had relatively smaller polyps of 2–5 mm in diameter. Third, CRP and PCT were significantly elevated within several hours.

Lee et al*.* further discussed three possible mechanisms of PPF [5, 8]. The first is that PPF may be a mild form of PPCS that develops by transmural burn. With the exception of abdominal tenderness, PPF is similar in terms other clinical symptoms and risk factors to PPCS. Notably, transmural burn in the colon wall is significant in both PPF and PPCS, thus suggesting that both might be generated by the same mechanism. PPF and PPCS are initiated by different degrees of transmural burn, with or without actual intestinal perforation. The second is that gut bacteria may translocate to the bloodstream via mucosal wounds during the colonoscopic procedure. The incidence of transient bacteremia was approximately 4% within 10 min after polypectomy. Contamination by enteric bacteria is inevitable, even when a disinfected colonoscope, sterile needles and sterile injection fluid are used during colonoscopy [9–11]. For instance, the propofol formulation for intravenous administration may be a possible contamination factor [12]. The third is that PPF may be attributable to an inflammatory mechanism other than infection. In general, polyps induce an inflammatory microenvironment with inflammatory cell infiltration and elevated proinflammatory cytokines, such as IL-6 and TNF-α. Thus, determining whether the patients with PPF developed fever because of the colonoscopy or the polypectomy itself is difficult.

As previously reported, seven patients developed PPF, of which two cases had no polyps, and one case had polyps 10–30 mm in diameter [5]. This finding suggests that colonoscopic examination without colonoscopic polypectomy also affects the intestinal bacteria or causes minimal intestinal-barrier damage. The patients with PPF in our study had multiple relatively small polyps (2–5 mm in diameter), in contrast to the previously reported findings. Although the causes of PPF are complicated, we believe that gut bacteria are translocated to the bloodstream via mucosal wounds.

PPCS, a rare and serious complication of colonoscopic polypectomy, results from an electrocoagulation injury to the bowel wall during polypectomy, which induces a transmural burn and localized peritoneal inflammation without clinical evidence of perforation on radiographic examination [13]. Within hours to 5 days after colonoscopic polypectomy, patients develop fever and other symptoms, including leukocytosis, localized abdominal pain and localized peritoneal signs.

In summary, this study may increase clinical awareness regarding PPF after colonoscopy. Early recognition and antibiotic therapy are critical, which can improve patient prognosis and avoid severe outcomes.

## Data Availability

All information about the patient come from department of Gastroenterology, Songjiang District Central Hospital. The data used and analyzed during the current study are included in this article.

## Abbreviations

PPF: Postpolypectomy fever
PPCS: Postpolypectomy electrocoagulation syndrome
GICU: General intensive care unit
CRP: C-reactive protein
PCT: Procalcitonin
WBC: White blood cell count
APC: Argon plasma coagulation
